# Effects of the ABC pathway on clinical outcomes in very elderly Chinese patients with atrial fibrillation. A report from the optimal thromboprophylaxis in elderly Chinese patients with atrial fibrillation (ChiOTEAF) registry

**DOI:** 10.1007/s11739-025-03928-0

**Published:** 2025-04-27

**Authors:** Ameenathul Mazaya Fawzy, Agnieszka Kotalczyk, Yutao Guo, Yutang Wang, Gregory Y. H. Lip, ChiOTEAF Registry Investigators

**Affiliations:** 1 Liverpool Centre for Cardiovascular Science, University of Liverpool and Liverpool Heart and Chest Hospital, Liverpool, United Kingdom; 2 Department of Cardiology, Congenital Heart Diseases and Electrotherapy, Medical University of Silesia, Silesian Centre for Heart Diseases, Zabrze, Poland; 3 Department of Pulmonary Vessel and Thrombotic Disease, Sixth Medical Centre, Chinese PLA General Hospital, Beijing, 100142 China; 4 Department of Cardiology, Second Medical Centre, Chinese PLA General Hospital, Beijing, 100853 China; 5 Department of Clinical Medicine, Aalborg University, Aalborg, Denmark

**Keywords:** Atrial fibrillation, ABC pathway, Holistic care, Management, Elderly, Prognosis

## Abstract

The current Atrial fibrillation Better Care (ABC) pathway for holistic or integrated management of AF is associated with improved clinical outcomes; however, data on the very elderly (aged ≥ 85 years) are sparse.

To evaluate the impact of ABC pathway on clinical outcomes amongst very elderly AF patients over a follow-up period of 1 year.

The ChiOTEAF registry is a prospective, multicenter nationwide study conducted from October 2014 to December 2018. Endpoints of interest were the composite outcome of all-cause death/any thromboembolism (TE), all-cause death, TE events, and major bleeding.

The eligible cohort included 1215 individuals (mean age 88.5 ± 3.3; 33.5% female), of which 142 (11.7%) were managed accordingly to the ABC pathway. ABC compliance was independently associated with lower odds of the composite outcome (odds Ratio (OR): 0.23; 95% confidence interval (CI): 0.08–0.66) and all-cause death (OR: 0.22; 95% CI: 0.07–0.75), without a significant increase in major bleeding compared to ABC non-compliance. Health-related quality of life (QOL) was also significantly higher in the ABC compliant group compared to the non-compliant group (EQ score 0.83 ± 0.17 vs. 0.78 ± 0.20; *p* =  0.004). Independent predictors of ABC non-compliance were prior major bleeding, chronic kidney disease, and dementia.

Our findings suggest that adherence to the ABC pathway in very elderly patients is associated with significantly improved survival and health-related QOL.

**Supplementary Information:**

The online version contains supplementary material available at 10.1007/s11739-025-03928-0.

## Introduction

Atrial fibrillation (AF) is the most common arrhythmia worldwide, increasing in prevalence with the ageing population. It is associated with high rates of mortality and morbidity and poses a substantial socioeconomic burden for healthcare systems. There are several factors combined, that could alleviate this through improved patient care.

The Atrial fibrillation Better Care or ABC pathway where A = Avoid stroke; B = Better symptom control; and C = Cardiovascular risk factor and Comorbidity management [1] is such an approach that encompasses holistic and integrated management of AF. The ABC pathway has been validated in multiple studies and there is ample evidence supporting its use in the management of AF patients [2]. The most recent AF-CARE pathway acronym recommended by the 2024 European Society of Cardiology guidelines, although non-validated, is essentially based on the components of the ABC pathway [2–6]. The latter has been recommended in several guidelines globally given its strong association with improved clinical outcomes in various cohorts worldwide [2, 7–10]. Although this is the case, data on regional and racial disparities in AF management are still scarce [11–13]. Moreover, data on the very elderly (aged ≥ 85 years) are limited, and the ‘real world’ population‐based benefit of ABC pathway compliance has not been previously evaluated in this high-risk population.

Herein, we assessed the implementation of ABC pathway and its impact on clinical outcomes amongst the very elderly (aged ≥ 85 years) Chinese patients enrolled in a nationwide prospective cohort study.

## Methods

The protocol of The Optimal Thromboprophylaxis in Elderly Chinese Patients with Atrial Fibrillation (ChiOTEAF) registry [14], as well as implementation of the ABC pathway overall [15] and characteristics of very elderly patients [16] have been previously described. Briefly, the study was conducted between October 2014 and December 2018 in 44 sites from 20 Chinese provinces; enrolling consecutive AF patients (with documented AF episode within 12 months prior to enrolment) presenting to cardiologists, neurologists, or surgeons. Follow-up visits were performed at 6- and 12-month and then annually for the next 2 years. Data were gathered by local investigators at enrolment and follow-up visits (including patient visits and/or chart reviews and/or telephone follow-ups) and reported into an electronic form.

### Ethics statement

The registry was approved by the Central Medical Ethics Committee of Chinese PLA General Hospital, Beijing, China (approval no S2014-065-01) and local institutional review boards. Written informed consent was obtained from all participants included in the study.

### Definitions

The ABC pathway was retrospectively evaluated according to its original definition, as follows: patients fulfilled the ‘*A*’ criterion if appropriately treated with oral anticoagulants (OACs) according to their TE risk; the ‘*B*’ criterion if they had true symptom control defined by an EHRA score of I or II (no symptoms or mild symptoms) at the baseline visit; and the ‘*C*’ criterion if they were managed with disease‑specific treatment(s) according to current guidelines at baseline. Patients were considered *ABC-adherent* when all three criteria (*A* + *B* + *C*) were achieved. The ‘very elderly’ were defined as patients aged ≥ 85 years [17].

Outcomes assessed and their definitions matched the EURObservational Research Programme Atrial Fibrillation (EORP-AF) Long-term General Registry [18]. The CHA_2_DS_2_-VASc [19] and the HAS-BLED scores [20] were used to assess the thromboembolic (TE) and bleeding risks. Bleeding events (intracranial and extracranial bleedings) were categorised based on the International Society on Thrombosis and Haemostasis (ISTH) definition [21]. Multimorbidity was defined as the presence of ≥ 2 comorbidities (in addition to AF) at enrolment amongst patients with AF [22, 23].

### Objectives

The primary objective of the present analysis was to evaluate the impact of ABC pathway on clinical outcomes at 1 year amongst very elderly patients with all available data to assess ABC management. The endpoints of interest were (i) the composite outcome of all-cause death/any TE (ischaemic stroke, transient ischaemic attack, or peripheral embolism), (ii) all-cause death, (iii) TE events, (iv) major bleeding, and (v) health-related quality of life (QOL) at 1 year. The secondary objectives were: (i)) to identify potential predictors of the ABC-adherent management in very elderly AF patients.

### Statistical analysis

Continuous variables were reported as mean ± standard deviation (SD); between-group comparisons were made using the Student’s *t* test or the Mann–Whitney U test (based on distribution). Categorical variables as counts and percentages; between-group comparisons were made by *χ*^2^ test or Fisher’s exact test (if required). Logistic regression analysis assessed the association between the ABC compliance and clinical outcomes (composite outcome of all-cause death/any TE, all-cause death, TE events, and major bleeding) amongst very elderly AF patients; adjusted for clinically significant variables (age, heart failure, prior ischaemic stroke, chronic kidney disease, chronic obstructive pulmonary disease). For the secondary objective, a logistic regression analysis was used to determine the predictors of ABC compliance in the very elderly group. Results were expressed as odds ratios (OR), 95% confidence intervals (CI), and *p* values. In all analyses, a *p* value < 0.05 was considered statistically significant. Statistical analysis was performed using SPSS^®^ version 24 (IBM Corp, Armonk, NY).

## Results

**Fig. 1:**
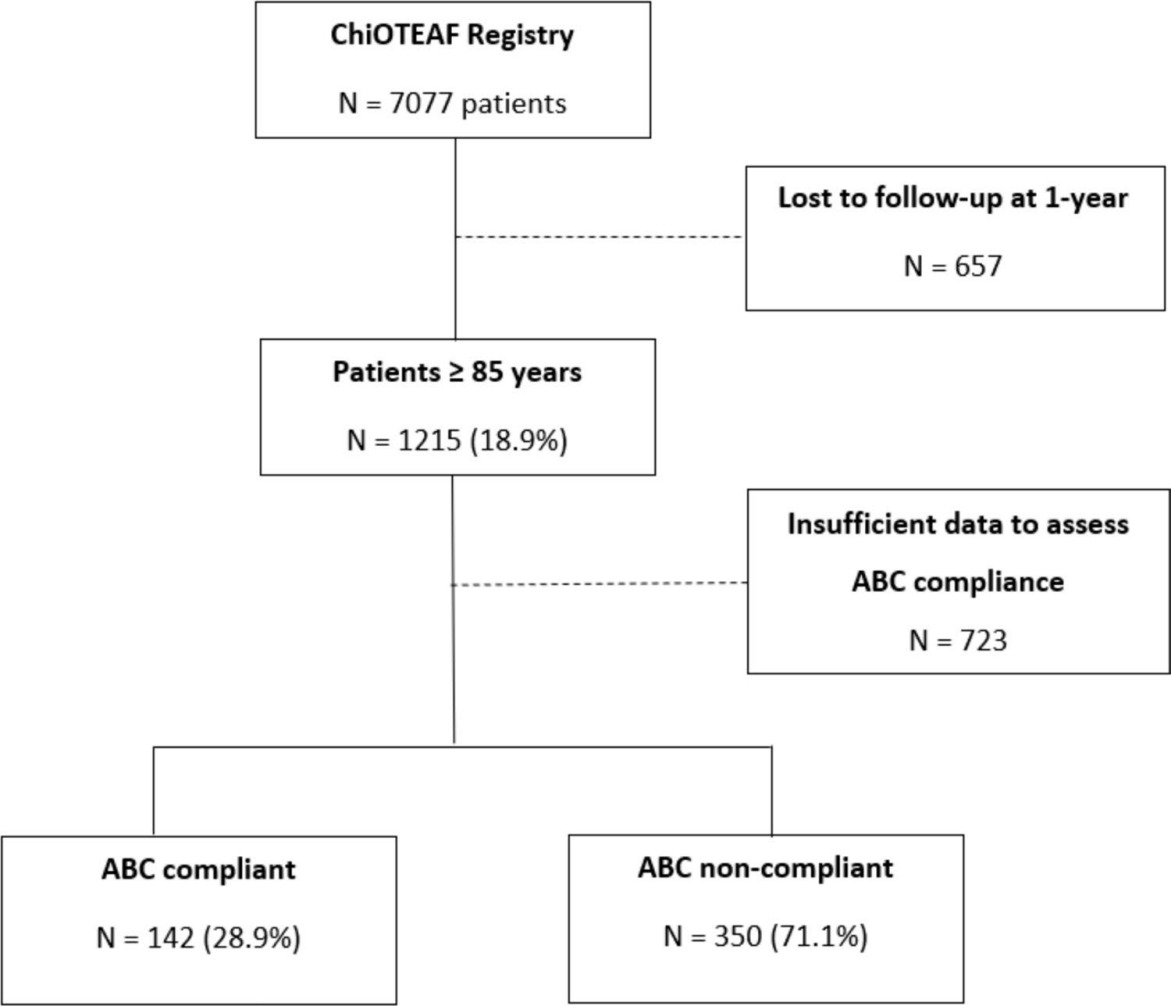
Flowchart of patient inclusion

The ChiOTEAF registry enrolled 7077 patients, of whom 657 (9.3%) were lost to follow-up at 1 year (Fig. 1). Among patients aged ≥ 85 years (*n* = 1215; mean age 88.5 ± 3.3 years, 30.5% female), only 492 (40.5%) had complete data to assess ABC pathway management. Of these, 142 (28.9%) were managed in accordance with the ABC pathway (ABC compliant group) and 350 (71.1%) were not managed according to it (ABC non-compliant group). Baseline characteristics are reported in Table 1.

**Table 1:** Baseline characteristics of the study cohort

	Overall elderly *N* = 1215 *n* (%)	ABC compliant *N* = 142 n (%)	ABC non-compliant *N* = 350 *n* (%)	*p*
Age*; years	88.5 ± 3.3	87.9 ± 2.5	88.2 ± 3.0	0.193
Female gender	370 (30.5%)	52 (36.6)	119 (34.0)	0.580
First-diagnosed AF	217 (22.9%)	22 (15.7)	86 (25.3)	0.022
BMI*; kg/m^2^	23.2 ± 3.6	23.5 ± 3.8	23.1 ± 3.6	0.220
Medical history				
Diabetes mellitus (*n* = 1209)	370 (30.6%)	47 (33.1)	101 (28.9)	0.353
Hypertension (*n* = 1209)	883 (73.0%)	114 (80.3)	253 (72.3)	0.065
Heart failure (*n* = 1209)	644 (53.3%)	74 (52.1)	184 (52.6)	0.926
Coronary artery disease (*n* = 1209)	791 (67.5%)	99 (69.7)	224 (64.0)	0.226
Prior ischaemic stroke	473 (38.9%)	49 (34.5)	117 (33.4)	0.819
Chronic kidney disease	301 (24.8%)	24 (16.9)	88 (25.1)	0.048
Liver disease	55 (4.5)	8 (5.6)	9 (2.6)	0.092
COPD (*n* = 1210)	271 (22.4%)	22 (15.5)	70 (20.0)	0.245
Sleep apnoea	50 (4.2%)	5 (3.5)	16 (4.6)	0.806
Dementia (*n* = 1210)	154 (12.7%)	7 (4.9)	33 (9.4)	0.105
Prior intracranial bleeding	52 (4.3%)	2 (1.4)	13 (3.7)	0.250
Prior extracranial bleeding	77 (6.4%)	7 (5.0)	13 (3.7)	0.530
Fall risk (*n* = 1209)	903 (74.7)	105 (73.9)	257 (73.4)	0.907
Current anaemia (*n* = 1210)	344 (28.4)	29 (20.4)	89 (25.4)	0.239
Multimorbidity (*n* = 1209)	1081 (89.4)	136 (95.8)	307 (87.7)	0.007
CHA_**2**_**DS**_**2**_VASc* (n = 1062)	4.6 ± 1.5	4.8 ± 1.4	4.5 ± 1.5	0.031
HAS-BLED* (n = 1064)	2.8 ± 1.1	2.6 ± 1.1	2.8 ± 1.0	0.041
Smoking status (*n* = 1183)				
-Former smoker	280 (23.7)	27 (19.9)	71 (20.9)	0.845
-Active smoker	32 (2.7)	5 (3.7)	7 (2.1)	0.337
AF management				
Oral anticoagulation (n = 1212)	320 (26.4%)	142 (100.0)	21 (6.0)	< 0.001
– VKA	115 (35.9%)	37 (26.1)	9 (2.6)	< 0.001
– NOAC	205 (64.1%)	105 (73.9)	12 (3.4)	< 0.001
Antiplatelet (n = 1210)	642 (53.1%)	22 (19.7)	232 (66.3)	< 0.001
Amiodarone (*n* = 1209)	75 (6.2%)	11 (7.7)	28 (8.0)	0.918
Propafenone (*n* = 1209)	23 (1.9%)	3 (2.1)	11 (3.2)	0.766
Digoxin (*n* = 1208)	170 (14.1%)	20 (14.1)	44 (12.6)	0.668
β-blockers (n = 1208)	662 (54.8%)	102 (71.8)	187 (53.7)	< 0.001
AF ablation (n = 1212)	12 (1.0)	6 (4.2)	4 (0.9)	0.020
CIED (*n* = 1212)	165 (13.6)	26 (18.3)	56 (16.0)	0.533

^*^ Mean ± standard deviation

AF—atrial fibrillation; BMI—body mass index; CHA_2_DS_2_VASc—Congestive heart failure/left ventricular dysfunction, Hypertension, Age ≥ 75 [doubled], Diabetes, Stroke [doubled], Vascular disease, Age 65–74, female Sex; CIED—cardiovascular implantable electronic device; COPD—chronic obstructive pulmonary disease; HAS-BLED—hypertension, abnormal renal/ liver function, stroke, bleeding history or predisposition, the labile international normalized ratio [INR], elderly, drugs/alcohol use; ICD—implantable cardioverter defibrillator; NOAC—non-vitamin K oral anticoagulant; VKA—vitamin K antagonists

Patients in the ABC compliant group were less likely to have chronic kidney disease (16.9% vs. 25.1%; *p* = 0.048) and first-diagnosed AF (15.7% vs. 23.3%; *p* = 0.022) but had a higher prevalence of multimorbidity (95.8% vs. 87.7%; *p* = 0.007) compared with the ABC non-compliant patients. No differences in age, sex, or other comorbidities were found between groups; however, the CHA_2_DS_2_VASc score was higher in the ABC compliant group (4.8 ± 1.4 vs. 4.5 ± 1.5).

With regard to the individual components of the ABC pathway, only 21 (6.0%) patients were compliant with the ‘*A*’ component whereas 337 (96.3%) and 274 (78.3%) of the 350 patients in the ABC non-compliant group fulfilled the ‘*B*’ and ‘*C*’ components respectively (Table S1). 2 (0.6%) patients did not meet any of the components of the ABC pathway, 64 (18.3%) met 1 component and 284 (81.1%) patients met 2 components. Of the 329 patients who were not on anticoagulation, 53 (16.1%) patients were deemed unsuitable for OAC by a physician despite the stroke risk, 118 (35.9%) were unwilling to take any OAC and 228 (69.3%) were on antiplatelet therapy. Fifty six (17.0%) patients were not accounted for at all, i.e. they were not unwilling to take OAC, were not on an ‘alternative treatment’ such as an antiplatelet agent and not deemed unsuitable for OAC. Given that 94% of patients were not on OAC, and the proportions of patients that were non-compliant with the ‘*B*’ and ‘*C*’ components were much smaller, it is likely that the outcomes were largely driven by non-compliance with the ‘*A*’ component.

### AF management and quality of life

In the ABC group, all patients were treated with an OAC (compared with 6.0% in the ABC non-compliant group) and the vast majority (73.9%) was treated with a non-vitamin K antagonist (NOAC). In contrast, 66.3% in the ABC non-compliant were treated with an antiplatelet agent. A higher proportion of patients in ABC compliant group had prior AF ablation (15.6% vs. 4.4%; *p* < 0.001). Health-related QOL was also higher in this group compared with the ABC non-compliant group (EQ score 0.83 ± 0.17 vs. 0.78 ± 0.20; *p* = 0.004) but no significant differences in EHRA scores were found.

### Clinical outcomes

Amongst patients managed according to the ABC pathway, a lower incidence of the composite outcome (2.8% vs. 12.0%; *p* = 0.001) and all-cause death (2.1% vs. 9.7% *p* = 0.002) were observed compared with the ABC non-compliant group. Odds of the composite outcome (OR: 0.23; 95% CI: 0.08–0.66) and all-cause death (OR: 0.22; 95% CI: 0.07–0.75) were lower in the ABC-managed patients. No significant differences in the incidence of TE and major bleeding events were evident between the groups (HAS-BLED score: 2.6 ± 1.1 vs. 2.8 ± 1.0 in the ABC compliant vs non-compliant group) (Table 2).

**Table 2:** The effects of ABC compliance on clinical outcomes (composite outcome; all-cause death; any thromboembolism; major bleeding) in very elderly patients

Outcomes	ABC compliant *N* = 142 *n* (%)	Non-ABC compliant *N* = 350 *n* (%)	*P*	Odds ratio* (95% CI)
Composite outcome#	4 (2.8)	42 (12.0)	0.001	0.23 (0.08–0.66)
All-cause death	3 (2.1)	34 (9.7)	0.002	0.22 (0.07–0.75)
TE events (*n* = 490)	1 (0.7)	10 (2.9)	0.189	0.26 (0.03–2.11)
Major bleeding (*n* = 490)	5 (3.5)	4 (1.1)	0.129	3.56 (0.91–13.99)

^*^Adjusted for age, heart failure, prior ischaemic stroke, chronic kidney disease, chronic obstructive pulmonary disease

^#^ Composite outcome of all-cause death/any thromboembolism

TE—thromboembolism; CI—confidence interval

### Exploratory analysis

#### Predictors of ABC pathway compliance

On multivariate analysis, (i) hypertension (OR: 1.68; 95% CI: 1.08–2.61) was positively associated with ABC pathway compliance amongst very elderly AF patients; whereas (ii) prior major bleeding (OR: 0.19; 95% CI: 0.06–0.61), chronic kidney disease (OR: 0.61; 95% CI: 0.38–0.97); and dementia (OR: 0.42; 95% CI: 0.19–0.92) were independently associated with lower odds of ABC compliance (Table 3).

**Table 3:** Predictors of the ABC compliance in very elderly patients with atrial fibrillation

	Univariate			Multivariate		
	Odds ratio	95% CI	*P*	Odds ratio	95% CI	*P*
Age	0.93	0.88–0.99	0.017	–	–	–
Female gender	1.37	0.95–1.98	0.090			
Diabetes mellitus	1.14	0.79–1.66	0.493			
Hypertension	1.58	1.02–2.44	0.040	1.68	1.08–2.61	0.021
Heart failure	0.95	0.67–1.35	0.769			
Coronary artery disease	1.24	0.85–1.81	0.271			
Prior ischaemic stroke	0.81	0.56–1.16	0.251			
Prior major bleeding	0.17	0.05–0.55	0.003	0.19	0.06–0.61	0.005
Chronic kidney disease	0.58	0.37–0.93	0.022	0.61	0.38–0.97	0.035
Liver disease	1.30	0.60–2.82	0.501			
COPD	0.60	0.38–0.97	0.037	–	–	–
Sleep apnoea	0.83	0.33–2.14	0.705			
Fall risk	0.96	0.64–1.43	0.828			
Dementia	0.33	0.15–0.71	0.005	0.42	0.19–0.92	0.030

ABC—Atrial fibrillation better care, CI—confidence interval; COPD—chronic obstructive pulmonary disease

## Discussion

In this study examining the ABC pathway in very elderly patients ≥ 85 years, we found that (i) only 29% of patients were managed using an integrated care approach; (ii) management according to the ABC pathway was associated with a significant reduction in all-cause death as well as the composite outcome of all-cause death and any TE event; (iii) ABC-adherent management was associated with a significantly better health-related QOL (iv) prior major bleeding, chronic kidney disease and dementia were independently associated with and were significant barriers to ABC non-compliance.

We also found no significant differences in the risk of TE and major bleeding events between the two groups. However, analyses of these outcomes were based on few events resulting in wide confidence intervals and results require verification in further large-scale studies. Nonetheless, our study demonstrates that adherence to the ABC pathway is associated with a mortality benefit even at 1-year and even in a multimorbid cohort with several complexities including an elevated bleeding risk.

In terms of baseline characteristics evaluated, a notable difference between the two groups was the significantly higher proportion of first-diagnosed AF patients in the ABC non-compliant group (25.3% vs. 15.7%, *p* = 0.022). Several studies have in fact demonstrated that first-diagnosed AF patients are less likely to be prescribed an OAC and instead, an antiplatelet agent [24, 25]. In a recent analysis from the BALKAN-AF registry, similar findings were observed with factors such as paroxysmal AF, prior bleeding and treatment in a non-emergency centre negatively associated with anticoagulant use, with variations depending on the geographical region [24]. Indeed, first-diagnosed AF (when patients were younger) may have led to a more aggressive anticoagulation strategy.

Another UK-based study reported outpatient referral for anticoagulant initiation as a reason for poor uptake in newly diagnosed patients whereas a Chinese study identified age > 80 years and discharge from a non-cardiology department as iatrogenic factors influencing OAC prescription in inpatients discharged with a diagnosis of AF [25, 26]. Elderly patients are more likely to be admitted under general medicine or geriatric wards rather than specialist cardiac wards. These findings demonstrate that although OAC under-prescription and aspirin substitution are reducing in prevalence, further efforts need to be undertaken, to improve this more and identify barriers at a local and regional level.

We found that 66.3% in the ABC non-compliant group were treated with an antiplatelet agent in our study, despite a lack of evidence supporting its use for stroke prevention. In a subgroup analysis of the AVERROES trial on older patients ≥ 85 years, apixaban compared to aspirin was associated with an absolute rate of stroke and systemic embolism of 1%/year versus 7.5%/year, hazard ratio [HR] 0.14 (95% CI 0.02–0.48) with no significant difference in major haemorrhage between the two drugs as well as between younger and older patients [27].

Our study identified prior major bleeding, chronic kidney disease and dementia as independent predictors of *ABC* non-compliance, though the likelihood is that they primarily influence the ‘*A*’ component of this pathway, with major bleeding the main concern. Whilst this is a valid consideration, particularly in the absence of randomised controlled trials (RCTs) and clinical guidelines specifically addressing these issues in the older population, several observational studies have demonstrated a net clinical benefit (NCB) of anticoagulation in this cohort. Nonetheless, bleeding remains a concern amongst healthcare professionals when prescribing antithrombotic therapy in Asia, often leading to suboptimal management [28].

In a study on very elderly Taiwanese patients ≥ 90 years old Chao et al. [29] demonstrated that anticoagulation in AF patients was associated with a positive NCB compared to treatment with antiplatelet agents or no treatment. Moreover, the risk of intracranial haemorrhage was lower in patients treated with NOACs compared to those treated with warfarin. 19.4% treated with warfarin and 23.4% treated with NOACs, had a prior history of major bleeding [29]. In contrast, analysis of the ≥ 85 years population from the START2-Register that compared VKAs with NOACs demonstrated a similar rate of bleeding between the two but a lower TE rate with VKAs, albeit the lower mortality rate with NOACs [30]. In a recent RCT, those over the age of 80 years old who would otherwise be ineligible for normal doses of anticoagulation due to reasons such as prior bleeding were randomised to receive either a lower dose of edoxaban 15 mg or placebo. A significant reduction in the risk of stroke was observed with the former, without any significant increases in the risk of major bleeding [31]. Similar findings were observed in a sub-analysis of the ELDERCARE-AF trial [32].

While dementia is deemed a non-modifiable factor for anticoagulant associated bleeding in AF, AF has been shown to double the risk of dementia owing to strokes which result in cerebral infarction. That said, dementia has been observed even in those who are stroke-free due to phenomena such as microemboli that cause cerebral hypoperfusion. Thus, OAC is crucial to mitigate this risk. Studies to date demonstrate similar substantial benefits of stroke risk reduction with OAC, without significant haemorrhagic complications, in patients with dementia, even in those with frailty, falls and low time in therapeutic range (when treated with VKAs) [33, 34]. Further, OAC therapy has been shown to reduce the risk of dementia by 16–40% and that of cognitive impairment by 26% by mediating the risk of ischaemic strokes [33, 35]. Retrospective assessment of the ABC pathway on a Korean AF population demonstrated a 20% reduction in the risk of dementia with ABC adherence [36].

Although the ABC pathway has not specifically been examined in very elderly patients, analyses thus far have demonstrated favourable clinical outcomes, even in high-risk patients including those with frailty and multimorbidities [37–39]. Whilst these are not necessarily synonymous with being elderly, findings from these as well as the present analysis will aid decision-making processes in older, multimorbid patients with clinical complexities. Indeed, this is a strong argument for appropriate characterisation and clinical evaluation of the patient with AF [40].

### Limitations

The main limitation of the ChiOTEAF registry is its observational character. The ChiOTEAF registry was aimed at analysing the relationship between the general management of AF and cardiovascular events at 6 months and 12 months, and not specifically designed to determine the role of ABC pathway adherent care on patients’ prognosis. ABC pathway adherence was assessed retrospectively, based on its definition published in 2017 and implementation in guidelines [3–5]. A moderate proportion of patients were lost to follow-up (9.3%) and only 40.5% of the very elderly had ABC pathway assessed, which may be a potential source of bias. Furthermore, the number of adverse clinical events may have been underreported. The number reported for TE and major bleeding events over the 1-year period was small and consequently, conclusions cannot be drawn for these results. The results of this study on an Asian population may also not be applicable to other populations due to ethnic differences in the epidemiology and AF-related outcomes [41, 42] as well as socioeconomic indices and variations between healthcare systems. Finally, data on anticoagulation control or the use of traditional Chinese medicines were not available and could not be considered in the analysis.

## Conclusion

This analysis from a real-world registry shows that implementation of the ABC pathway is feasible in very elderly AF patients. Management according to the ABC pathway improves clinical outcomes and survival in very elderly Chinese patients. Further studies on this population are required to substantiate these findings.

## Supplementary Information

Below is the link to the electronic supplementary material.

Supplementary file1 (DOCX 20 kb)

## Data Availability

The datasets used and analysed during the current study are available from the corresponding author on reasonable request.

## Abbreviations

ABC: Atrial fibrillation better care
AF: Atrial fibrillation
CI: Confidence interval
OR: Odds ratio
TE: Thromboembolism
QOL: Quality of life

## Code availability

Statistical analysis was performed using SPSS® version 24 (IBM Corp, Armonk, NY).
